# Editorial: Peripheral Regulators of Obesity

**DOI:** 10.3389/fendo.2019.00357

**Published:** 2019-06-04

**Authors:** Andrew J. McAinch, Deanne H. Hryciw, Sudip Bajpeyi

**Affiliations:** ^1^Institute for Health and Sport, College of Health and Biomedicine, Victoria University, Melbourne, VIC, Australia; ^2^Australian Institute for Musculoskeletal Science, Victoria University, Melbourne, VIC, Australia; ^3^School of Environment and Science, Griffith Sciences, Griffith University, Nathan, QLD, Australia; ^4^Department of Kinesiology, College of Health Sciences, University of Texas at El Paso, El Paso, TX, United States

**Keywords:** obesity, diabetes, adipose tissue, body composition, TRPV1 - transient receptor potential vanilloid type 1 receptor, PPAR gamma, HIV, hepatocyte growth factor (HGF)

The incidence of obesity is increasing significantly worldwide and is considered one of the most significant public health challenges due to the associated disease states increasing morbidity and mortality and reducing quality of life. Despite the impact (both social and economic) of obesity little is known of the complex physiology that dictates the maintenance of the obese state and resistance to weight loss, and the link between obesity and complex diseases, including type 2 diabetes. Understanding the peripheral abnormalities that are present in obesity are essential to our development of treatments for these conditions. This special issue aimed to elucidate at least some of the complex interactions that occur particularly in the periphery. The special issue contained a mix of 4 review articles and 6 original manuscripts ranging from cell culture studies to clinical trials and large cross sectional observations.

The review by Christie et al. discusses TRPV1 structure and function, how this is linked to the endocannabinoid system, energy homeostasis, appetite control, and gastrointestinal vagal afferents. The review concluded with how TRPV1 activation may stimulate energy expenditure particularly via brown adipose tissue thermogenesis and its role in β-cell function in diabetes. However, despite TRPV1's potential roles in energy expenditure and blood glucose regulation, it has complex interactions with many signaling pathways. Thus further research to provide fundamental physiological understanding of TRPV1 to enable targeted treatment options for obesity and associated metabolic diseases is required.

Peroxisome proliferator-activated receptor γ (PPARγ) and its role in adipocytes via dynamic change of transcription was reviewed by Ma et al. PPARγ plays a key role in adipogenesis through ligand binding and selective interaction with transcriptional corepressors or coactivators. Of note, PPARγ plays a critical role in the determination of the brown and beige adipocytes. Ma et al. conclude via the discussion of the role of specific PPARγ phosphorylation sites and how stimulation of phosphorylation at different sites results in potentially different physiological outcomes.

The special issue continues with the review investigating insulin resistance induced by antiretroviral drugs in HIV-Patients by Pedro et al. This review discusses the increase in circulating proinflammatory cytokines and bacterial lipopolysaccharides resulting in downregulation of insulin signaling. Through discussion of what is known about the molecular signaling associated with antiretroviral induced insulin resistance as well as the link with gut microbiota and obesity and dyslipidemia, this review highlights the need for further research into this condition to enable the next generation of targeted medications to ensure the long term quality of life of HIV patients.

The theme of insulin resistance is also continued in the review by Oliveira et al. which discusses the role of hepatocyte growth factor (HGF) in insulin resistance and diabetes. This review discusses the structure and function of HGF and its role in the regulation of glucose metabolism in different cells and tissues. It then continues to provide insight into the involvement of HGF in inflammatory responses as well as cancer therapy and thus the complexity of targeting HGF in obesity or diabetes.

The reviews captured in this special issue provide an interesting snapshot of the current state of research into several signaling cascades that are associated with obesity and insulin resistance as well as the influence of other disease states on these signaling cascades in a number of tissues, including adipose tissue. The special issue then continues with a study by Merlin et al. which demonstrates that cultured white adipocytes derived from regions such as inguinal white adipose tissue (iWAT) with adequate sympathetic innervation respond efficiently to agonists, but only in combination with an additional priming stimulus such as rosiglitazone. This contrasts with *in vivo* iWAT depots in which chronic sympathetic tone is sufficient to maintain the capacity for brite (or beige) activation. This study emphasized the importance of activating both browning and thermogenic programs in cultured white adipocytes in order to reach maximum browning capacity.

Understanding the impact of not just obesity, but also how our body stores and distributes fat mass can have important implications on our health. Cui et al., determined that in Qingdao, China, being classified as overweight or having abdominal obesity as determined by waist circumference was associated with depression whereas waist-to-hip ratio was not. The impact of body composition not just body weight was also investigated by Colleluori et al. which observed a U-shaped distribution of fat mass when correlated to estradiol (Serum E_2_) levels in postmenopausal women which was different to the linear increase seen when only looking at body weight or body mass index (BMI). This study raises some speculations regarding E_2_ sensitivity and fat mass regulation in postmenopausal women. An inverse association with circulating SIRT1 and adiposity levels across the body weight range (underweight to obese) was also observed by Mariani et al. This inverse association was also associated with liver fat content, insulin and other markers of metabolic phenotype.

Even in the healthy weight range, according to BMI, Park et al. determined that young women classified as plump (around 55 kg) compared to slim (around 46 kg) young women showed more atherogenic features in LDL and HDL as well as elevated oxidation and glycation with loss of antioxidant ability and apoA-I. Despite these observations, interventions can improve the health outcomes, as determined in this special issue's final manuscript by Telles et al. In this study Telles et al. demonstrated an age effect, with women with central obesity between 30 and 45 years of age having a greater reduction in measures such as waist circumference and BMI following 12 weeks of yoga compared to the control group who just received nutritional advice. This reduction was not seen in the 46–59 year age range, which demonstrates that early interventions may have better health outcomes.

## Author Contributions

All authors listed have made a substantial, direct and intellectual contribution to the work, and approved it for publication.

### Conflict of Interest Statement

The authors declare that the research was conducted in the absence of any commercial or financial relationships that could be construed as a potential conflict of interest.

